# Axial morphology and 3D neurocranial kinematics in suction-feeding fishes

**DOI:** 10.1242/bio.036335

**Published:** 2018-09-15

**Authors:** Yordano E. Jimenez, Ariel L. Camp, Jonathan D. Grindall, Elizabeth L. Brainerd

**Affiliations:** 1 Department of Ecology and Evolutionary Biology, Brown University, 80 Waterman Street, Providence, RI 02912, USA; 2 Friday Harbor Laboratories, University of Washington, 620 University Road, Friday Harbor, WA 98250, USA; 3 School of Aquatic and Fishery Sciences, University of Washington, 1122 Boat Street, Seattle, WA 98105, USA

**Keywords:** Axial skeleton, Body shape, Pterygiophore, Supraneural, VROMM, XROMM

## Abstract

Many suction-feeding fish use neurocranial elevation to expand the buccal cavity for suction feeding, a motion necessarily accompanied by the dorsal flexion of joints in the axial skeleton. How much dorsal flexion the axial skeleton accommodates and where that dorsal flexion occurs may vary with axial skeletal morphology, body shape and the kinematics of neurocranial elevation. We measured three-dimensional neurocranial kinematics in three species with distinct body forms: laterally compressed *Embiotoca lateralis*, fusiform *Micropterus salmoides*, and dorsoventrally compressed *Leptocottus armatus*. The area just caudal to the neurocranium occupied by bone was 42±1.5%, 36±1.8% and 22±5.5% (mean±s.e.m.; *N=*3, 6, 4) in the three species, respectively, and the epaxial depth also decreased from *E. lateralis* to *L. armatus*. Maximum neurocranial elevation for each species was 11, 24 and 37°, respectively, consistent with a hypothesis that aspects of axial morphology and body shape may constrain neurocranial elevation. Mean axis of rotation position for neurocranial elevation in *E. lateralis*, *M. salmoides* and *L. armatus* was near the first, third and fifth intervertebral joints, respectively, leading to the hypothesis of a similar relationship with the number of intervertebral joints that flex. Although future work must test these hypotheses, our results suggest the relationships merit further inquiry.

## Abstract (precis)

**Summary:** Aspects of axial skeletal morphology and body shape correlate with 3D neurocranial motions in three species, generating hypotheses of how axial shape may impact cranial motions in fishes.

## INTRODUCTION

For many suction-feeding fish, neurocranial elevation is an important motion for expanding the buccal cavity and generating suction (e.g. Schaeffer and Rosen, 1961; Carroll and Wainwright, 2006; Camp and Brainerd, 2014; Van Wassenbergh et al., 2015). The neurocranium elevates by rotating dorsally about a transverse axis relative to the body. To produce this elevation, the vertebrae linked to the neurocranium must flex dorsally across one or more intervertebral joints (IVJs; this study includes the craniovertebral joint among the IVJs, unless otherwise noted). Therefore, it has been hypothesized that vertebral morphology is modified for permitting high degrees of neurocranial elevation in some fish (Lesiuk and Lindsey, 1978; Lauder and Liem, 1981; Huet et al., 1999). Despite this interest in vertebral specializations, few studies have explored how the axial skeleton as a whole may affect neurocranial motions and, conversely, how the requirement of neurocranial elevation may influence body shape and axial skeletal structures.

**Fig. 1.**
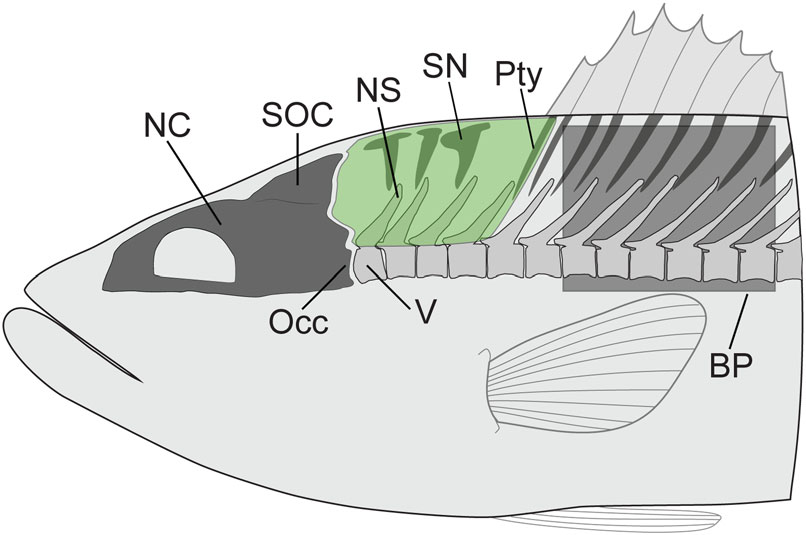
**Axial morphology and neurocranial elevation.** Lateral view of the axial skeleton in the region hypothesized to undergo dorsal flexion during neurocranial elevation. Shaded in green is the region used for taking bone area measurements. Illustration is based on the morphology of *Micropterus salmoides* in a resting position. BP, body plane; NC, neurocranium; NS, neural spine; Occ, occiput; Pty, pterygiophore; SN, supraneural; SOC, supraoccipital crest; V, Vertebra.

Body shape and the morphology of the axial skeleton have typically been studied in the context of lateral bending of the body for swimming (e.g. Gray, 1933; Hebrank et al., 1990; Jayne and Lauder, 1995; Westneat and Wainwright, 2001; Long et al., 2002; Porter et al., 2009; Nowroozi et al., 2012; Nowroozi and Brainerd, 2012, 2014; Moran et al., 2016). It is reasonable to expect that they are also relevant to dorsal bending of the body during suction feeding but few studies have examined them in this context. Without data on both 3D neurocranial kinematics and axial morphology, it is difficult to generate, let alone test, hypotheses of how axial and body morphology relate to neurocranial elevation. In this study, we examine some aspects of axial skeletal morphology (Fig. 1) and overall body shape together with 3D motions of the neurocranium. While ligaments, tendons and axial musculature also play important roles in vertebral column flexion, the present study focuses on the axial skeleton and overall body shape.

**Fig. 2.**
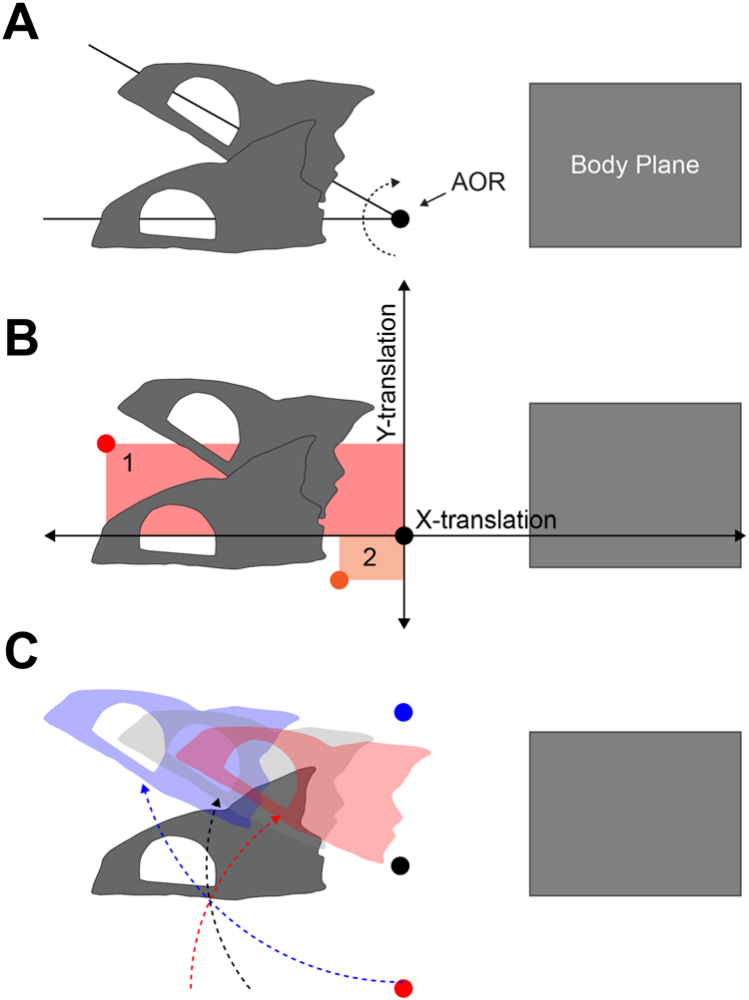
**Determining the** **AOR** **and kinematic trajectory of the neurocranium.** (A) The starting and final positions of neurocranial elevation. The intersection of the lines through the neurocranium marks the position of the axis of rotation (AOR). (B) Hypothetical JCS positions for measuring neurocranial motion. As shown by points 1 (red) and 2 (orange), dorsoventral and anteroposterior placement of a JCS affects measured X-and Y-translation values, respectively. Translation is reduced only when the JCS is positioned closer to the actual AOR and translation is zero at the AOR where motion is captured as a pure rotation. (C) Three examples of neurocranial elevation, with final neurocranium positions and AORs in corresponding colors, showing the kinematic significance of AOR position.

Axial morphology may be related to the position on the vertebral column where dorsal flexion is likely to occur during neurocranial elevation. This location can be described as the axis of rotation (AOR) between the neurocranium and the body of the fish. The AOR is the pivot point between the neurocranium and the body at which neurocranial elevation can be described as pure rotation without translation (Fig. 2A). As such, the AOR is the centre of rotation, with dorsal flexion of the IVJs likely distributed on either side of it (except in rare cases of a single IVJ specialized for dorsal flexion; see below). Several studies have estimated the position of the AOR using morphological observation, specimen manipulation and 2D kinematic analysis. Based on the manipulation of specimens, Gregory (1933) suggested that the AOR is located near the post-temporal-supracleithral joint, which Carroll et al. (2004) used as the AOR for calculating the mechanical advantage of epaxial musculature in centrarchid fishes. Others have suggested that the AOR is located at the craniovertebral joint based on its specialized morphology (Lauder and Liem, 1981), or in the case of *Rhaphiodon vulpinus,* Lesiuk and Lindsey (1978) implied that the AOR must be located in the IVJ just posterior to the Weberian apparatus, since the apparatus is relatively immobile. In other instances, the AOR has been determined in largemouth bass and pipefish based on modified versions of the Reuleaux method, which estimates the AOR from a 2D kinematic analysis of the position of the neurocranium at rest and at maximum elevation (Reuleaux, 1876; Van Wassenbergh et al., 2008, 2015).

One aspect of axial morphology proposed to relate to neurocranial elevation is the position and shape of the supraneurals, neural spines and pterygiophores (Fig. 1). As part of a larger study of epaxial muscle activity during suction feeding in largemouth bass, Thys (1997) studied the supraneurals, neural spines, and pterygiophores located just caudal to the neurocranium. X-ray images of largemouth bass postmortem showed these bones moving closer together during moderate cranial elevation of just 8° and Thys hypothesized that these bones could become crowded together in a way that would limit cranial elevation. Lesiuk and Lindsey (1978) also reported that neighboring neural spines restrict *ex vivo* dorsal flexion in *Rhaphiodon vulpinus*. Hence, if the supraneurals, neural spines and pterygiophores together occupy a greater area of the region immediately caudal to the neurocranium in some species relative to others, this densely-packed configuration might be associated with lower magnitudes of neurocranial elevation during suction feeding.

The morphology of the vertebrae and intervertebral joints may also influence the magnitude and the location of dorsal body flexion, as suggested by studies of fishes with extremely high (>45°) magnitudes of neurocranial elevation (Lesiuk and Lindsey, 1978; Lauder and Liem, 1981; Huet et al., 1999). Studies of *R. vulpinus* (Lesiuk and Lindsey, 1978)*, Luciocephalus pulcher* (Lauder and Liem, 1981) and *Uranoscopus scaber* (Huet et al., 1999) each noted that some vertebrae appear to be specialized for dorsal flexion. In particular, hypertrophied zygapophyses have been suggested to limit torsional (long-axis rolling) motions of the neurocranium to protect the spinal cord from injury during extreme dorsal flexion (Lauder and Liem, 1981). This suggests species with larger and more closely articulated zygapophyses may show less torsion (roll) of the neurocranium (Holmes, 1989), but neurocranial roll has so far been directly measured in only a single species, largemouth bass (Camp and Brainerd, 2014).

The potential effects of body shape are less clear. Large neurocranial elevations are possible across a range of body shapes as demonstrated by the laterally compressed *R. vulpinus*, the fusiform *L. pulcher* and the dorsoventrally compressed *U. scaber.* While body width and length could potentially relate to neurocranial kinematics, body height seems most likely to be related to neurocranial elevation. A tall body, with a tall supraoccipital crest and tall epaxial musculature, has been hypothesized to enhance the sub-ambient pressures generated during suction feeding by increasing the moment arm and physiological cross-sectional area for epaxial muscles (Carroll et al., 2004). But, for the same amount of neurocranial rotation, the dorsal tip of a taller supraoccipital crest will translate further caudally than that of a shorter crest. And more caudal motion of the supraoccipital crest may be limited or prevented by the supraneurals, neural spines, and pterygiophores (Fig. 1). Furthermore, the magnitude of cranial elevation in a deep-bodied fish may be limited by the overall bulk of tissue located just caudal to the head. Thus, the height of the body – and particularly the height of the epaxial region dorsal to the vertebral column – could theoretically be related to the magnitude of neurocranial elevation.

Our study presents a synthesis of 3D kinematics of the neurocranium, 3D measurement of the AOR, and comparative morphology of three distantly related species. Our goal is to generate more informed hypotheses about the relationships between axial skeletal morphology, body shape and neurocranial kinematics rather than to provide definitive tests of those hypotheses. We began with descriptions of the body shape and axial morphology of three species of suction feeders with a range of body shapes: laterally compressed and tall-bodied, *Embiotoca lateralis* (striped surfperch); fusiform, *Micropterus salmoides* (largemouth bass); and dorsoventrally compressed, *Leptocottus armatus* (Pacific staghorn sculpin). We then recorded 3D motion of the neurocranium and a body plane using XROMM (Brainerd et al., 2010) and VROMM (X-ray and Video Reconstruction of Moving Morphology, respectively). Using a body plane as a frame of reference, we measured 3D kinematics of the neurocranium and identified the position of the AOR for neurocranial elevation. Finally, we quantified and compared the accuracy of our 3D method with that of the 2D Reuleaux method, which has been previously used for locating the AOR.

## RESULTS

### Axial morphology

**Fig. 3.**
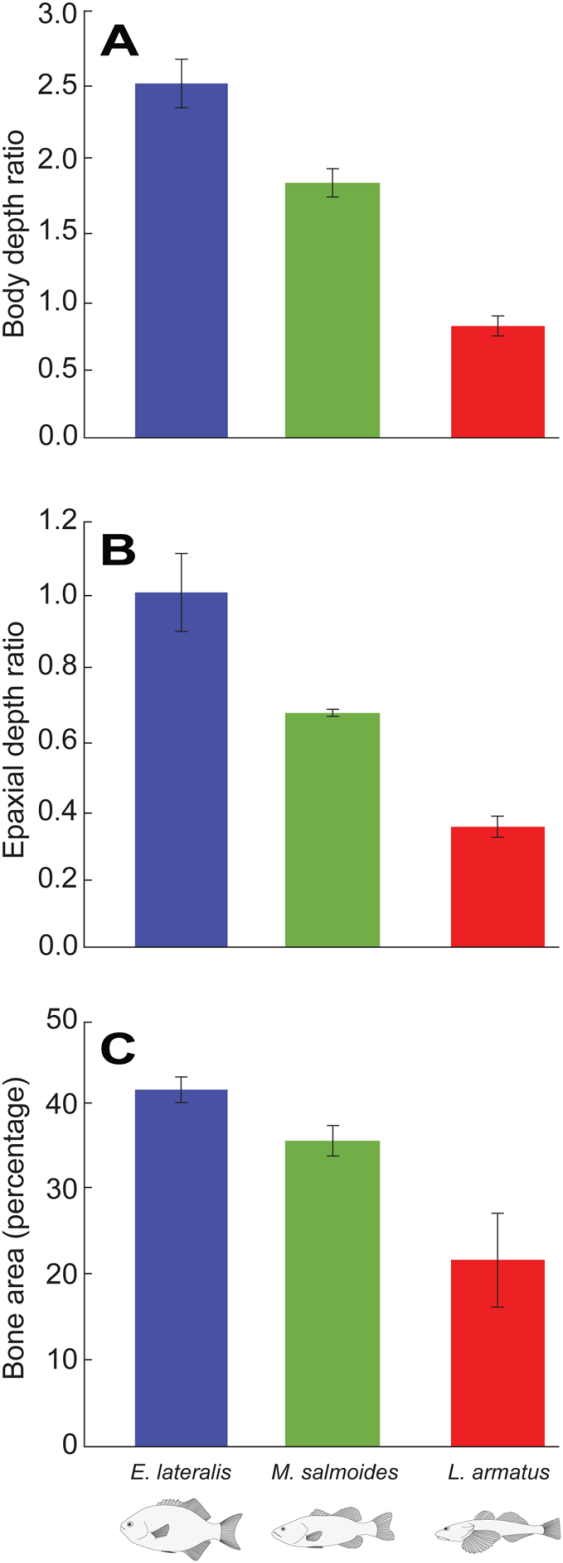
**Quantification of body form and bone area percentage in the region just caudal to the neurocranium (means±s.e.m.).** (A) Body depth measured as dorsoventral body height divided by width. (B) Depth of the epaxial area. (C) Area percentage occupied by bone in a 2D X-ray projection of the epaxial area between the neurocranium and the first dorsal pterygiophore.

**Fig. 4.**
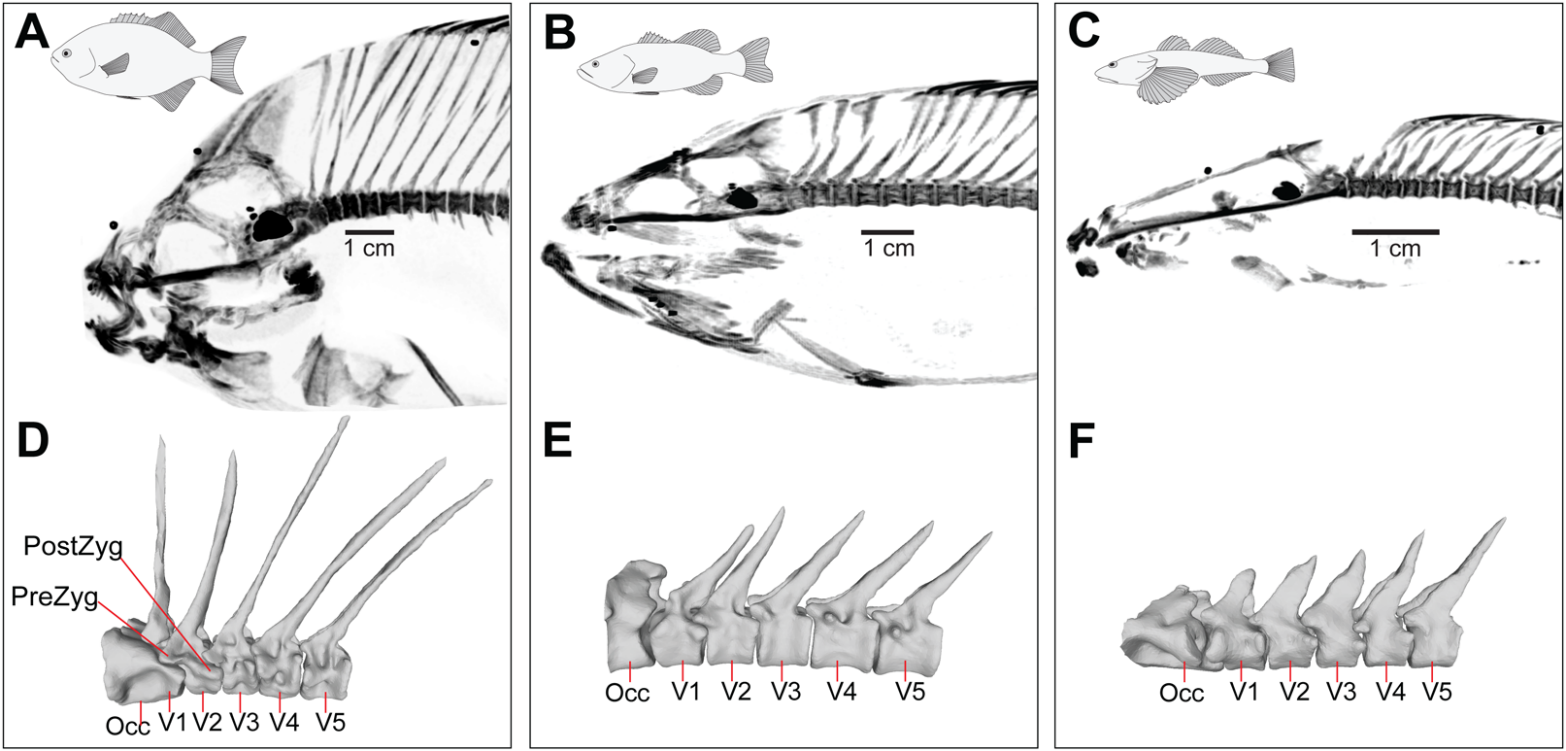
**Comparative axial morphology for three study species.** (A-C) Lateral view, CT volume renderings of (A) *E. lateralis*, (B) *M. salmoides* and (C) *L. armatus*. Images are maximum intensity projections with inverted gray levels to make X-ray positive images. (D-F) Mesh models of the first five vertebrae of (D) *E. lateralis*, (E) *M. salmoides* and (F) *L. armatus*. Mesh models were made from dissected and rearticulated vertebrae, and are shown in lateral view with cranial to the left. For A, a different contrast was applied to the supraoccipital crest, relative to the rest of the skeleton, to accurately show the bone in this area. Occ, occiput; PostZyg, post-zygapophysis; PreZyg, pre-zygapophysis; V1-V5, vertebrae 1 to 5.

The overall body shape of *E. lateralis* is tall and laterally compressed, with a height to width ratio of 2.5±0.17 (mean±s.e.m., *N*=3) for the whole body and 1.0±0.11 for the epaxial region (Fig. 3). The axial skeleton in the region just caudal to the head is comprised of long neural spines, supraneurals and pterygiophores (Fig. 4A). The first two supraneurals lie adjacent to the anterior aspects of the neural spines of V1 and V2, respectively, and the third supraneural is positioned posterior to the neural spine of V2. The neural spines from V2 and V3 form an open wedge, inside which the first pterygiophore and the ventral process from the third supraneural bone are positioned, effectively filling the space (Fig. 4A). The second pterygiophore lies adjacent to the posterior aspect of the V3 neural spine and the third and all subsequent pterygiophores nearly touch the anterior aspects of the neural spines. The first three vertebrae also have pre- and post-zygapophyses, with the exception of V1 which lacks pre-zygapophyses because it is fused with the occiput (Fig. 4D). Post-zygapophyses are robust and protrude from the lateral body of the centrum, crossing the joint and articulating inferior to the pre-zygapophysis of the subsequent vertebra. Posterior to V4, the vertebral centra are spool shaped and lack zygapophyses, with V4 being transitional between the vertebrae with and without zygapophyses (Fig. 4D).

The overall body shape of *M. salmoides* is fusiform, with a height to width ratio of 1.8±0.10 (mean±s.e.m., *N*=6) for the whole body and 0.66±0.01 for the epaxial region (Fig. 3). The fusiform body of *M. salmoides* has shorter neural spines and less overlap between the second and third neural spines and the supraneurals than in *E. lateralis* (Fig. 4B). The neural spines of V1 and V2 and the first two supraneurals lie in close apposition, but the remaining neural spines are more widely spread apart from each other. The first supraneural lies adjacent to the anterior aspect of the neural spine of V1, a pattern that is repeated for the two subsequent supraneurals (Fig. 4B). No supraneurals or pterygiophores fill the space between the neural spines of V3 and V4. The first and second pterygiophores fill the space between the neural spines of V4 and V5, just as the third and fourth pterygiophores fill the space between the neural spines of V5 and V6. Starting with the fifth pterygiophore, only one pterygiophore inserts between each pair of neural spines. Like *E. lateralis*, the zygapophysial joints start at V1 and begin to decrease in size around V4 (Fig. 4E).

The body of *L. armatus* is dorsoventrally compressed with a height to width ratio of 0.79±0.07 (mean±s.e.m., *N*=4) for the whole body and 0.34±0.03 for the epaxial region (Fig. 3). Unlike the other two species, it lacks supraneural bones entirely, resulting in a gap between the neurocranium and the first pterygiophore (Fig. 4C). Neural spines and pterygiophores overlap and contact each other in a similar manner to *M. salmoides*. The first and second pterygiophores surround the anterior and posterior aspects of the neural spine of V3, whereas the remaining pterygiophores are evenly distributed between neural spines. In *L. armatus*, zygapophysial joints are present from V1 to V5 (Fig. 4F), though these vertebrae are not associated with any distinct supraneural arrangement like *E. lateralis* and *M. salmoides*. V5 is transitional for vertebrae with articulating zygapophyses and vertebrae with non-articulating zygapophyses. Vertebrae with non-articulating zygapophyses have relatively larger intervertebral spaces (spaces between the vertebral centra) than the more cranial joints (Fig. 4C).

The bones in the area just caudal to the supraoccipital crest of the three species occupy different proportions of the available space (Fig. 3C). From the images, the neural spines, supraneurals and pterygiophores appear to be most densely packed in *E. lateralis* (Fig. 4A), followed by *M. salmoides* with substantial spaces between the supraoccipital crest and the first supraneural and between the third supraneural and first pterygiophore (Fig. 4B). Supraneurals are absent in *L. armatus*, making this species appear to have the most open space behind its head (Fig. 4C). To quantify these observations, we measured the area percentage occupied by bone and found a mean (±s.e.m.) of 42±1.5% in *E. lateralis,* 36±0.8% in *M. salmoides* and 22±5.5% in *L. armatus* (Fig. 3C). One-way ANOVA with species as the effect showed a significant overall effect of species on percentage of bone (*P*<0.0001) and Tukey pairwise post-hoc tests showed that all three species were significantly different from each other (*P*<0.05). These quantitative results confirm that the neural spines, supraneurals and pterygiophores are most densely packed in *E. lateralis,* followed by *M. salmoides* and then *L. armatus.*

### 3D cranial kinematics and the AOR

**Fig. 5.**
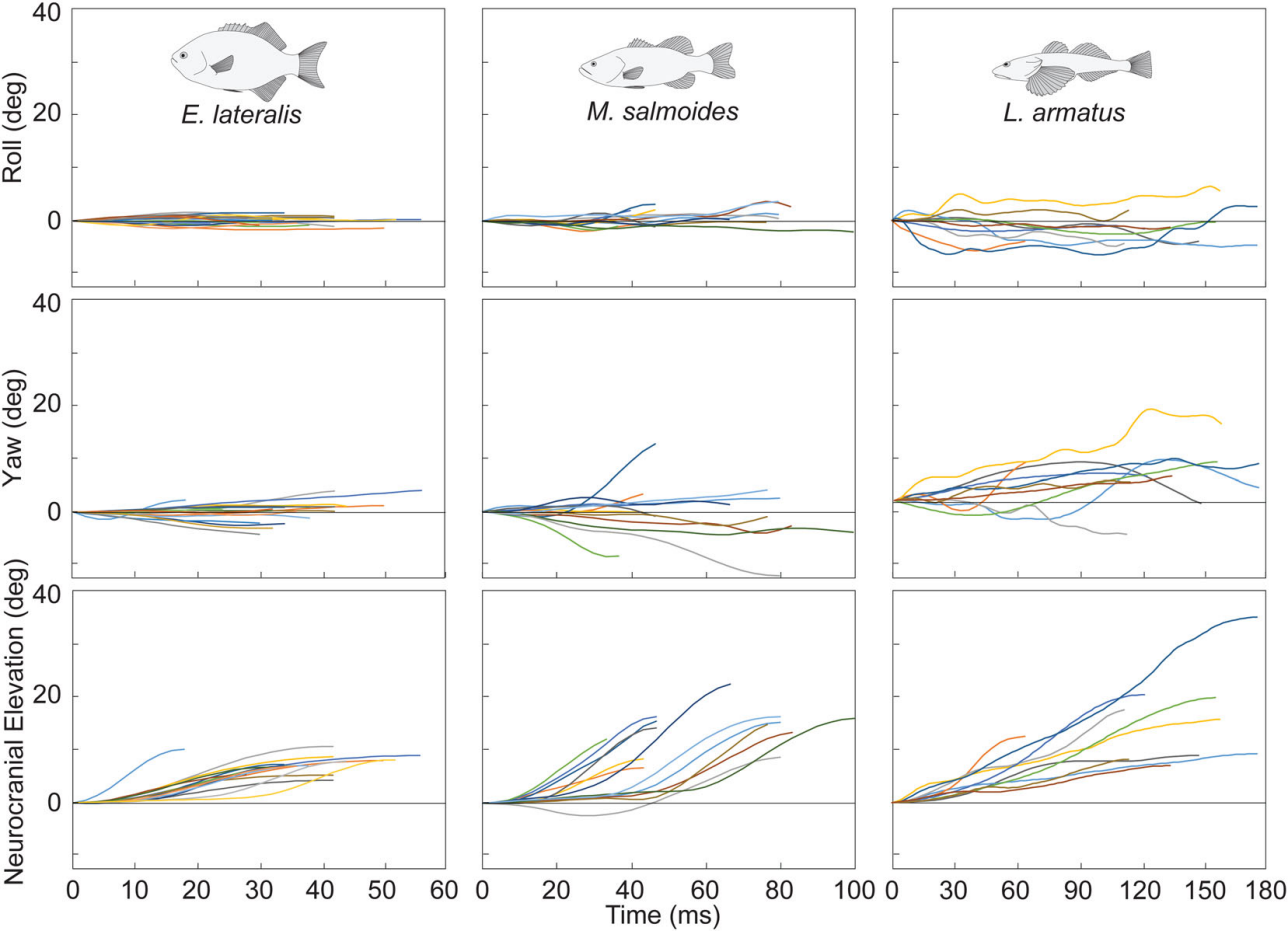
**Kinematic profiles of the neurocranium, relative to the body plane, during the expansive phase.** In each graph, each line represents rotation during a single strike. Lines are color coded by strike, so each strike has the same color for all rotations. Positive roll indicates clockwise rotation of the head (from an anterior view). Positive yaw indicates the head turning to the left (from a dorsal view). Positive neurocranial elevation denotes upward head lifting. The Y-axis of each graph is scaled the same throughout, whereas the X-axis is scaled differently for each species, with time 0 indicating the frame before the onset of neurocranial elevation.

All three species used neurocranial elevation as part of their suction strikes (Fig. 5). As mentioned above, *L. armatus* sometimes showed no neurocranial elevation or even neurocranial depression; only strikes that used neurocranial elevation were included for *L. armatus*. Although 3D neurocranial kinematics of the *M. salmoides* strikes have been reported previously (Camp and Brainerd, 2014), in that study they were measured using a joint coordinate system (JCS) positioned at the craniovertebral joint. In this study, we report the 3D neurocranial kinematics of these strikes as measured by a JCS placed at the AOR, in order to make them directly comparable to those of *L. armatus* and *E. lateralis*.

Neurocranial kinematics were measured with a JCS placed at the AOR during the expansive phase of each strike; from the onset to the peak of neurocranial elevation. Means (±s.e.m.) for maximal neurocranial elevation were 7.9±0.7° for *E. lateralis* (*N*=4 individuals), 14.4±2.2° for *M. salmoides* (*N=*3 individuals) and 11.2±2.8° for *L. armatus* (*N=*4 individuals). Nested ANOVA, with individuals nested within species, yielded a significant overall model (*P*<0.0001) with significant effects of both individuals (*P*=0.015) and species (*P*=0.00046). Tukey pairwise post-hoc tests showed that maximal neurocranial elevation was statistically significantly higher in *M. salmoides* than in *E. lateralis* (*P*<0.05), although *L. armatus* was not significantly different from either of the other two species (*P*>0.05). Mean (±s.e.m.) duration of the expansive phase was 37.9±3.3 ms for *E. lateralis*, 63.0±9.9 ms for *M. salmoides* and 125.4±13.3 ms for *L. armatus*. The maximum observed neurocranial elevation across all strikes for each species was 11.1° for *E. lateralis*, 23.8° for *M. salmoides* and 36.5° for *L. armatus*.

In addition to elevating, the neurocranium in all three species yawed and rolled relative to the body plane (Fig. 5). Cranial yaw denotes the magnitude and direction that the fish turned its head to the left or right relative to the body during the strike (positive is to the fish's left, negative to the right). Means for the magnitude (absolute value) of maximal yaw were 1.7±0.2° for *E. lateralis*, 5.0±1.1° for *M. salmoides* and 6.3±1.2° for *L. armatus*. Nested ANOVA yielded a significant overall model (*P*=0.0025) with a significant effect of species (*P*=0.0025) but not individual (*P*=0.44). Tukey post-hoc tests showed that maximal yaw in *M. salmoides* and *L. armatus* was not statistically significantly different (*P*>0.05), although both had significantly higher yaw than *E. lateralis* (*P*<0.05).

Cranial roll denotes the magnitude and direction that the fish rotated its head about its long axis (Fig. 5). Means for the absolute magnitude of maximal roll were 0.9±0.1° for *E. lateralis*, 2.1±0.3° for *M. salmoides* and 3.5±0.9° for *L. armatus*. Nested ANOVA yielded a significant overall model (*P*<0.0001) with significant effects of species (*P*<0.0001) and individuals (*P*=0.05). Tukey post-hoc tests showed that maximal roll was significantly different between all three pairs of species (*P*<0.05).

**Fig. 6.**
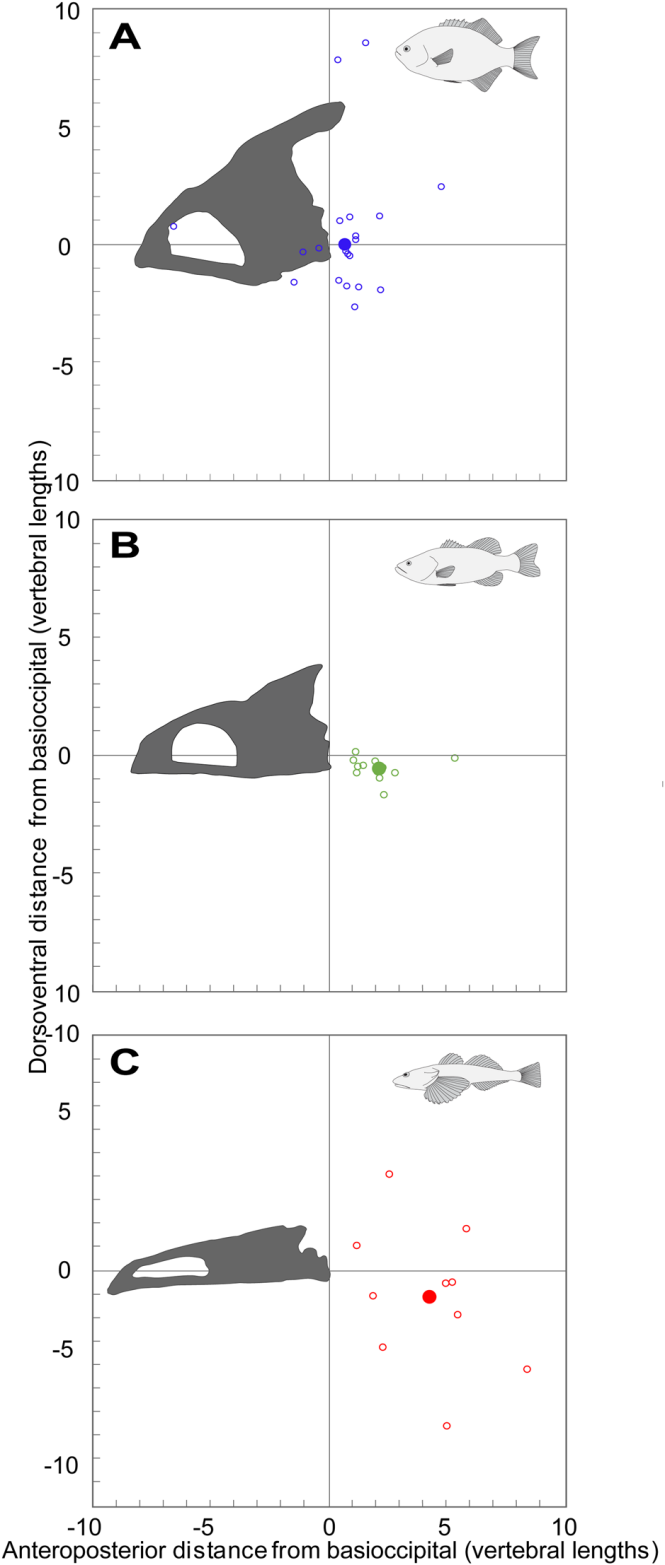
**Normalized** **AOR** **position for each species.** The position of the AOR for each strike (open circles) is plotted as the dorsoventral and anteroposterior distances (normalized to vertebral centrum length) of the AOR from the occiput. (A) *E. lateralis*, (B) *M. salmoides*, and (C) *L. armatus*. The occiput is the origin (0, 0), with positive X-and Y-coordinates denoting caudal and dorsal positions, respectively. For each species, the average AOR position is shown as a large, filled circle. AOR was measured with the 3D JCS method (Fig. 7B).

The mean AOR for neurocranial elevation was located closest to the occiput (craniovertebral joint) in *E. lateralis*, more caudally in *M. salmoides* and most caudally in *L. armatus* (Fig. 6). The AORs in all three species were distributed across a wide range of positions (Fig. 6). In *E. lateralis*, most AORs were clustered around the occiput, though two strikes had AORs in positions far dorsal to the vertebral column. Close examination and re-estimation of AORs for these two outliers reaffirmed the measured position and yielded no methodological explanation for the far dorsal position. The AORs in *M. salmoides* were clustered between the second and fourth IVJs, posterior to those of *E. lateralis*, and were almost always at the dorsoventral level of the vertebral column. Finally, AORs in *L. armatus* were widely and sparsely distributed across the anteroposterior and dorsoventral axes.

**Fig. 7.**
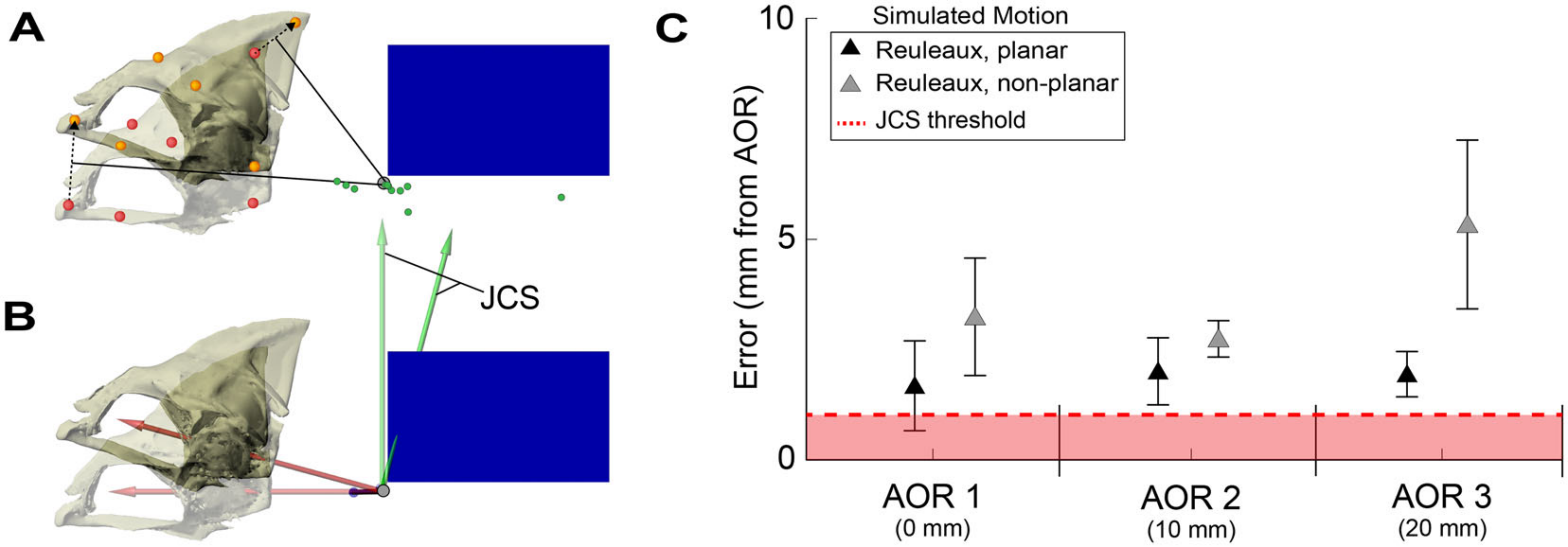
**Comparison of 2D and 3D methods for locating the AOR, using simulated planar and non-planar neurocranial motion.** (A) The 2D Reuleaux method uses changes in landmark position (red and orange circles) to estimate the true AOR (gray circle). Each landmark is connected by a line (black dashed arrows), which is then bisected by a perpendicular line (black solid lines). The intersection of any two perpendicular lines is the estimated AOR position. Pairwise combinations of landmarks generate a total of 15 AOR estimates (green circles). (B) The 3D method creates multiple joint coordinate systems (JCSs) to identify the position that minimizes translation values. (C) Both methods were used to calculate the AOR from simulations of both planar (black triangles) and non-planar (gray triangles) neurocranial elevation at three distances posterior to the occiput. Error for the Reuleaux method was calculated as the mean (±s.e.m) linear distance between the 15 estimated AORs and the actual AOR. Maximum error of the 3D method is equal to the JCS sampling density, 1 mm for these simulations.

## DISCUSSION

We found significant differences in the area percentage of bone occupying the space behind the head in our three species (Fig. 3C), but our expectation that less bone area and shorter epaxial height would correlate with more neurocranial elevation was only partially borne out. Mean neurocranial elevation was higher in *M. salmoides* than in *E. lateralis*, as expected, but *L. armatus* produced a highly variable amount of neurocranial elevation, such that the mean magnitude was not significantly different from either of the other two species (Fig. 5). However, if we consider the maximum magnitude of cranial elevation observed for each species across all strikes, the pattern does match our expectations with bone area decreasing in the order *E. lateralis, M. salmoides, L. armatus* (Fig. 3) and maximum observed neurocranial elevation increasing in the order *E. lateralis* (11.1°), *M. salmoides* (23.8°) and *L. armatus* (36.5°). The large magnitude of neurocranial elevation in *L. armatus* was accompanied by the absence of supraneurals and the enlarged intervertebral spacing posterior to V5 (Fig. 4C). Conversely, *E. lateralis* had the tallest body and epaxial ratio (Fig. 3A,B) and an axial skeleton with long interdigitating bones (Fig. 4A), along with the least amount of neurocranial elevation. These data suggest that axial skeleton morphology may indeed be related to the magnitude and location of dorsal flexion of the vertebral column. However, the data presented in this study are correlative and *in vivo* measurements of the kinematics of the axial skeleton are needed to test this hypothesis. Furthermore, claims regarding maximal performance must be made cautiously, as Astley et al. (2013) have previously pointed out, these claims are more easily falsified than verified. Despite our attempts to elicit maximal performance, it is possible that such strikes did not occur in this study and that our study species can produce greater amounts of neurocranial elevation than we observed.

The anteroposterior position of the AOR for neurocranial elevation varied among our study species, with the mean AOR located closest to the craniovertebral joint in *E. lateralis*, farther back in *M. salmoides* and most caudal in *L. armatus* (Fig. 6). In some instances, *E. lateralis* and *L. armatus* had AORs outside the body. Similarly, Van Wassenbergh et al. (2008) found that when producing neurocranial elevation, pipefish typically have AORs located outside the body. The mean AORs in *E. lateralis* and *M. salmoides* were located close to the area occupied by supraneurals; very few strikes had AORs in the region with interdigitating neural spines and pterygiophores. Conversely, only some AORs in *L. armatus* were found in the space where supraneurals are absent, and the majority occurred in the region with neural spines and pterygiophores. Interestingly, that is the region where the spacing of the intervertebral joints (IVJ) appears to be relatively larger in *L. armatus* than in the other species (Fig. 4C). These patterns suggest there could be a relationship between vertebral flexion and the distribution of the IVJs, neural spines, supraneurals, and pterygiophores.

Despite producing similar mean amounts of neurocranial elevation, *M. salmoides* and *L. armatus* had very different AOR positions. This shows that kinematic similarities (i.e. magnitude of neurocranial elevation) do not necessarily indicate similar patterns of dorsal flexion in the IVJs. Conversely, *M. salmoides* and *E. lateralis* may be flexing a similar number of IVJs, but it is likely that *M. salmoides* is flexing them to a greater extent to produce more neurocranial elevation than *E. lateralis*. The more posterior AORs in *L. armatus* suggest that they typically distribute dorsal flexion across more IVJs than *E. lateralis* and *M. salmoides*.

In some fish, neurocranial elevation has been thought to be primarily the product of flexion at a single joint, based on the specialized appearance of the surrounding vertebrae (Lesiuk and Lindsey, 1978; Lauder and Liem, 1981). Others have suggested that multiple joints flex to produce a smooth curve during dorsal flexion in stargazers (Huet et al., 1999) and still others surmised that the first four to five vertebrae are involved (Liem, 1978). While our study does not definitively resolve this issue, there is good reason to think that the species we studied exhibit the latter condition. With the exception of *E. lateralis*, nearly all strikes have AORs posterior to the craniovertebral joint position, and we suggest that flexion is distributed across multiple joints, especially in strikes that have more caudal AORs. The alternative is that all flexion occurs at a single joint. However, this scenario is biologically implausible since it would require neurocranial elevation – which often exceeds 10° – to be the sole product of dorsal flexion at a single IVJ. The degree of IVJ dorsal flexion during feeding has not been quantified, although Nowroozi and Brainerd (2013) used marker-based XROMM to quantify axial skeletal motion during the startle response of *Morone saxatilis*. They found that dorsal flexion of the IVJs is usually under 5° and that lateral flexion rarely exceeds 10° in the cervical region, so it seems unlikely that dorsal flexion can exceed 10°. Finally, even if a single joint could produce all the dorsiflexion for neurocranial elevation, some mechanism would still be needed to restrict the motion of the surrounding joints that are otherwise mobile.

### Yaw and roll

Rotation about the Y-axis of our JCS (green axis in Fig. 7B) measures yaw of the head relative to the body and can result from lateral flexion of the body in the region of the body plane, as well as yaw at the IVJs near the cranium. All three species sometimes exhibited substantial amounts of yaw, with maximum values exceeding 50% of those for neurocranial elevation of the strike (Fig. 5). Neurocranial yaw has a more variable kinematic profile than neurocranial elevation because it is directional (i.e. fish can use left and right yaw). This directionality reflects how yaw may be used for repositioning the mouth during prey capture, although this was not measured in our study. Being able to yaw may allow predators to adjust their strike and place elusive prey within the suction flow field or directly inside the mouth (Muller et al., 1982; Ferry-Graham et al., 2003; Day et al., 2005; Higham et al., 2006). While it is possible that differences in the ability to yaw and aim place constraints on feeding behavior and performance, the contributions of lateral flexion to suction feeding are likely only of secondary importance – unlike the clear role of lateral flexion in locomotion (Jayne and Lauder, 1995; Westneat and Wainwright, 2001).

Rotation about the X-axis of our JCS (red axis in Fig. 7B) measures roll of the head about its long axis relative to the body. Because neurocranial roll does not appear to confer any advantage to suction feeding, we were surprised by the amount of cranial roll detected, although of the three cranial rotations measured, it still had the lowest magnitudes for each species (Fig. 5). Cranial roll is possibly a byproduct of simultaneous neurocranial elevation and yaw, which may destabilize the body and produce superfluous motion in the form of axial torsion. This destabilizing effect may explain why roll in *L. armatus*, which produces the most motion across the most joints, is so high. We predicted that more well-developed zygapophyses may act to prevent roll but we found the opposite correlation in our three study species. We observed the most roll in *L. armatus*, which had distinct zygapophyses on the first five vertebrae (Fig. 4F), less roll in *M. salmoides* with zygapophyses on the first four vertebrae (Fig. 4E) and the least roll in the species with zygapophyses on just the first three vertebrae, *E. lateralis* (Fig. 4D). It appears that other features, such as overall body form, may influence the amount of cranial roll more than the number of zygapophyses.

### Advantages and limitations of VROMM

VROMM shows promise as an alternative to XROMM for measuring 3D skeletal kinematics without X-ray imaging, but only for situations where external markers can be rigidly attached to the bones, as is the case in the dermal bones of ray-finned fishes with tightly attached skin. The overall marker tracking precision for this study was 0.122 mm, compared with 0.1 mm or better for XROMM (Knörlein et al., 2016). Several factors account for the lower precision of VROMM, including refraction, external marker motion and calibration object design. Because VROMM uses standard (not X-ray) cameras, images can be distorted by the refraction that occurs at the air-tank-water interface. This is especially true when cameras are positioned more obliquely to the tank, since higher angles of incidence increase refraction and may reduce precision (Henrion et al., 2015).

We found lower precision for *L. armatus* than *E. lateralis* (0.182 versus 0.104 mm), which may have been caused by greater effects of refraction and by greater marker motion. The effect of refraction is likely to be greater when the animal moves across the tank during a strike, perhaps explaining why trials were less precise for *L. armatus* – which tended to use more body ram – than for *E. lateralis*. It is also possible that skin deformations and whole-body movements caused the external markers to move, especially in *L. armatus*, which had looser skin.

### Accuracy of 2D and 3D methods for measuring AOR

The JCS method was generally more accurate than the Reuleaux method when analyzing non-planar motions. Optimizing the accuracy of the Reuleaux method requires that certain conditions be met. For example, two landmarks should never form – or come close to forming – a straight line with the AOR (Fig. 7A). This can result in high errors, since near-parallel lines must extend for longer distances before intersecting. It follows that landmark placement relative to the AOR requires careful consideration when using the 2D method, but this can only be accomplished if the AOR position is assumed *a priori*. Therefore, obtaining high errors with this method becomes more likely when we consider the high variability of AOR positions among and within species (Fig. 6).

The Reuleaux method also requires a high level of confidence that the motion is planar, since non-planar motions increase error (Fig. 7C). One way this issue is commonly avoided is by limiting analysis to footage where the camera is aimed perpendicular to the animal. But this should be done cautiously, as there can be instances when motion is non-planar even when perpendicular views are acquired. While tradeoffs exist between the Reuleaux and JCS methods (e.g. between ease of use and accuracy), they share the assumption that there is a single AOR. This assumption is reasonable, especially for the original purpose of the Reuleaux method, which was to describe the kinematics of machines consisting of rigid parts with constrained motions (Reuleaux, 1876). However, since biological motion is not always so uniform and constrained, the AOR may change dynamically throughout a behavior. For the purposes of this study, finding the overall AOR is useful for approximating where dorsal flexion may occur along the vertebral column. Finally, the JCS method doubled as a tool for both locating the AOR and measuring 3D neurocranial motion, the latter of which the Reuleaux method cannot do.

### Concluding remarks

Correlating kinematics and the AOR for neurocranial elevation with axial skeletal morphology is valuable for exploring the role of the axial skeleton during suction feeding. The magnitude of neurocranial motion can approximate the degree of dorsal flexion the axial skeleton must accommodate and the AOR can approximate where that flexion is likely to occur. A more extensive comparison of diverse morphologies and a more quantitative analysis of *in vivo* skeletal motion may uncover the relationship between body shape, axial morphology and neurocranial kinematics.

Our data show that neurocranial motions during suction feeding are not only 3D, but that interspecific variation can exist in all dimensions. Neurocranial motions are made possible by flexion in the cervical region, making their motions neck-like. Since yaw and roll were sensitive to directionality (Fig. 5), these values likely represent only half the total flexibility of the vertebral column, such that a mean absolute value of 5° yaw indicates a total range of motion of 10° (i.e. from left to right). The cervical vertebrae in *Morone saxatilis* undergo substantial amounts of lateral flexion during some C-starts (Nowroozi and Brainerd, 2013), further supporting the idea that fish cervical vertebrae are not only distinct in their morphology and mechanical properties, but also in their motions. Given this added complexity, it is unclear how the axial morphology may be optimized for both feeding and swimming motions and whether functional tradeoffs are made. This study underscores the importance of studying post-cranial morphology to understand suction feeding fully.

## MATERIALS AND METHODS

### Animals

We collected *Leptocottus armatus* (Girard, 1854) and *Embiotoca lateralis* (Agassiz, 1854) in the San Juan Islands, Washington, USA. Fish were housed at University of Washington, Friday Harbor Labs in a flow-through seawater system. Fish were fed shrimp and squid daily, which were also used in feeding trials. We collected VROMM data for *E. lateralis* (*N=*4; 197, 201, 210 and 213 mm SL) and *L. armatus* (*N=*4; 125, 210, 292, and 316 mm SL), and re-analyzed XROMM animations of *Micropterus salmoides* (Lacépède, 1802; *N=*3; 201, 228, and 233 mm SL) originally published in Camp and Brainerd, 2014. The University of Washington's Institutional Animal Care and Use Committee (IACUC) approved all collections, husbandry, and experimental procedures.

### Morphology

We CT (computed tomography) scanned three to six individuals from each species. In addition to the three *M. salmoides* used for XROMM, we scanned three more individuals (200, 248 and 255 mm SL) for morphological examination only. We scanned *M. salmoides* at 0.625 mm slice thickness (Philips Medical System, Best, The Netherlands). Scans for one *E. laterali*s and one *L. armatus* were made at 0.185 mm slice thickness or lower (Animage FIDEX, Pleasanton, USA). Scans for the other three *E. lateralis* and *L. armatus* were made at 0.142 mm slice thickness or lower (Microphotonics Skyscan 1173, Allentown, USA). Using Horos CT-imaging software (version 1.1; 64-bit; www.horosproject.org), we rendered and examined the morphology of the axial skeleton, including the shape, orientation, and spacing of bones. We were unable to find a CT window width and height that showed the thin lamina of bone that fills the supraoccipital crest of *E. lateralis* while also providing suitable contrast for the rest of the skeleton. Therefore, we applied different contrast to the supraoccipital crest in this species to correctly reflect the presence of bone in this area. To examine vertebral morphology closely, we dissected out the first five vertebrae of one individual from each species. We CT scanned the disarticulated vertebrae for higher resolution models of the morphology on the Animage FIDEX scanner at 0.162 mm slice thickness or lower.

From CT cross-sections we measured the height and width of the body and the height and width of the epaxial region. Longitudinal body position was standardized by selecting a slice at the anteroposterior position of the second rib. For the epaxial region, we measured the height of the body dorsal to the vertebral centrum and width across the body at the dorsoventral level of the centrum.

From the CT scans we also measured the area percentage occupied by bone in the epaxial region just caudal to the head (area outlined in Fig. 1). The MIP (maximum intensity projection) tool in Horos was used to create a sagittal thick slice spanning the mediolateral width of the vertebrae and view the MIP of the slice from the lateral side. We excised an image of the area bounded by the caudal aspect of the neurocranium, the vertebral column, the caudal aspect of the first pterygiophore and the dorsum of the fish. In ImageJ (1.51, Wayne Rasband, NIH), we thresholded the image to include all bone within the threshold area and measured the area percentage of the whole image occupied by bone (i.e. neural spines, supraneurals and first pterygiophore). To test reproducibility of this method we repeated the whole process three times on the same CT scan for each species, starting with a new MIP in Horos through to measurement in ImageJ, and found a maximum variation of ±1% of area in the measurements (e.g. bone occupying 25±1% of 2D projected area behind the head).

### VROMM

**Fig. 8.**
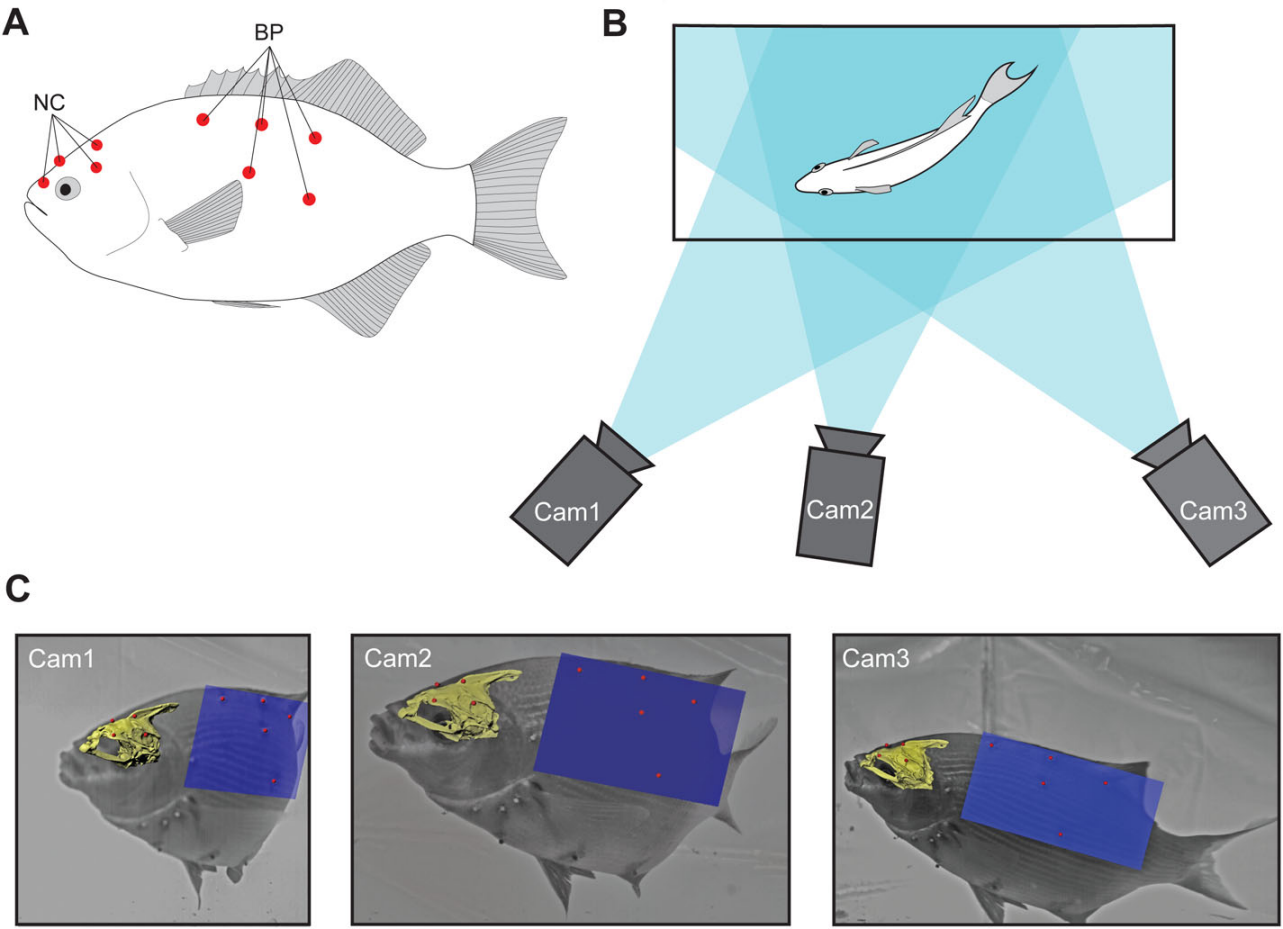
**Methods for VROMM videography.** All images are from *E. lateralis*. (A) Lateral view, generalized marker attachment sites for the neurocranium (NC) and body plane (BP). (B) Dorsal view, camera positions used for feeding trials. Blue shading shows each camera view, with the darkest area showing where the fish is visible from all three cameras (though only two camera views are needed). (C) High-speed video frames at mid-strike from all three camera views with VROMM-animated models of the neurocranium and body plane superimposed. Markers on the pectoral girdle and hypaxial region are visible but were not analyzed in this study.

For VROMM, we anesthetized specimens with MS-222 and sutured white plastic beads (1.8 mm diameter) onto the skin of the neurocranium (three to four beads) and body (four to six beads). Markers were placed as far as possible from each other in a non-linear arrangement (Fig. 8A) since 3D motion cannot be captured – i.e., one degree of freedom is lost – when markers are arranged in a straight line, wider marker distributions can more accurately capture its motion. After marker attachment, we returned the fish to its tank and allowed it to recover fully prior to starting feeding trials.

Three 1024 PCI Photron high-speed cameras (Photron USA Inc., San Diego, USA) recorded feeding events from three different perspectives (Fig. 8B,C). In some trials for *E. lateralis* we used video from just two cameras, which is sufficient for 3D reconstruction as long as the markers are visible in both cameras (Brainerd et al., 2010). We filmed all strikes at 500 Hz with the exception of three trials for *L. armatus*, which were filmed at 250 Hz. Before and after each set of feeding trials, we recorded images of a calibration object, made from Lego bricks with precise 3D locations, inside the tank. Using XMALab software (version 1.3.1; available from bitbucket.org/xromm/xmalab), we calibrated the tank space with images of the 3D calibration object and tracked the external markers to generate XYZ motion coordinates. The precision of tracking external markers suffers from non-circular marker projections (Knörlein et al., 2016) and from distortion produced by refraction when filming through multiple media (i.e. water and an aquarium; Henrion et al., 2015). Nonetheless, the mean standard deviation of intermarker distances for beads rigidly attached to the neurocranium for this VROMM study was 0.122 mm (based on the standard deviations for 150 pairwise distances from 31 strikes; 0.104 mm for *E. lateralis* and 0.182 mm for *L. armatus*) compared with 0.1 mm or less for XROMM (Brainerd et al., 2010; Knörlein et al., 2016).

To generate 3D motion data from individual markers, we created rigid bodies in XMALab, which uses the tracked XYZ coordinates of three or more markers to calculate their motion as a single rigid body with six degrees of freedom. Two rigid bodies were made, one for the neurocranium and one for a plane defining the position of the body, based on the four to six beads attached to the body (Fig. 8A). Body beads were attached to soft tissue and were expected to move slightly relative to each other and did not form a true rigid body. When we calculate ‘rigid body’ motion from four to six bead positions, the result is a 3D pose that averages across the positions of all of the beads (Camp and Brainerd, 2014). In this study, during any given strike, the pairwise distances between the body beads typically varied less than 0.2 mm and at most up to 1 mm.

Rigid body transformations (translations and rotations) were imported into Autodesk Maya 2014 (San Rafael, USA) to animate a polygonal mesh model of the neurocranium and a polygon plane object for the body plane (Fig. 8C). To generate a mesh model of the neurocranium for VROMM, we CT scanned the specimens with the markers still attached (see ‘Morphology’ section above for scan details). We created mesh models of the neurocrania of all individuals in Horos with additional mesh refinement in MeshLab (ISTI-CNR, Pisa, Italy). We also made mesh models of the first five vertebrae for anatomical study, although none of the vertebral structures were animated.

Given that our study includes data from separate XROMM and VROMM projects, different prey types, sizes and exclusion criteria were used. Prey were typically delivered directly in front of the fish, but the position of the food item relative to the mouth was highly variable (above, below, to a side), as it was often determined by how the fish approached the prey. For *M. salmoides*, individuals were recorded feeding on live goldfish (Camp and Brainerd, 2014). During most feeding trials for *L. armatus* and *E. lateralis*, individuals were recorded performing a single successful strike on a piece of shrimp or squid. However, in one trial, *L. armatus* was fed a small live shrimp, and in another trial, the same individual missed on the first strike and performed two additional strikes; only the first attempt was analyzed since it was the fastest of the three and involved substantial cranial expansion. We excluded slow strikes with minimal movement of the cranial elements (e.g. neurocranium, hyoid and lower jaw). Both *E. lateralis* and *M. salmoides* used neurocranial elevation for every strike. Conversely, *L. armatus* sometimes showed no neurocranial elevation or even neurocranial depression, particularly when taking food from the bottom of the aquarium. We only included strikes in which *L. armatus* showed neurocranial elevation, including some bottom strikes, resulting in seven useable strikes from one individual and just one acceptable strike per individual from the other three.

### Quantifying the AOR

To find the axis of rotation (AOR) we used joint coordinate systems (JCSs), implemented in Autodesk Maya with the XROMM Maya Tools (version 2.1.1; available from xromm.org/software). The XROMM and VROMM animations contained two rigid bodies: the neurocranium and the body plane (Fig. 8A). The body plane provided a fish-based frame of reference that moved with the fish but did not move as a part of cranial expansion. To identify the AOR during neurocranial elevation, we created JCSs and aligned them with the long axis of the fish as defined by the neurocranium and body plane. JCSs consist of proximal and distal anatomical coordinate systems (ACS). The proximal ACS was fixed to the body plane and the distal ACS followed the neurocranium, allowing the JCS to measure neurocranial motion as rotations and translations relative to the body plane.

JCSs were oriented so that Z-axis rotation represents neurocranial elevation (i.e. dorsal rotation), Y-axis rotation corresponds to cranial yaw (i.e. lateral rotation to the left or right) and X-axis rotation to cranial roll (i.e. long-axis rotation). All rotations and translations refer to motion of the neurocranium relative to the body plane, with zero rotation defined as the pose of the fish before the onset of neurocranial elevation in each strike and zero time as the frame before the onset of neurocranial elevation.

Each JCS at a different position generates different translation values, so we located the AOR by placing numerous JCSs at different anteroposterior and dorsoventral positions of the fish in 1 mm increments. This method is based on the principle that even a purely rotational motion can be measured as having translation, depending on the position of the JCS relative to the AOR (Fig. 2B). For example, if a JCS is placed anterior to the AOR, then the magnitude of Y-axis translation will increase in proportion to its distance from the AOR. Similarly, if the JCS is placed dorsal to the AOR, then X-axis translation will increase.

To find the anteroposterior position of the AOR for neurocranial elevation, we placed multiple JCSs in the animation space for each strike until a clear trend in Y-axis translation values revealed its position (Fig. 2B). We repeated this procedure for finding the dorsoventral position, but instead of sampling anteroposterior positions and minimizing Y-translation, we sampled dorsoventral positions and minimized X-translation. We quantified the position of the AOR as x- and y- coordinates of the distance (cm) from the occiput. These distances were then normalized to vertebral centrum lengths (using V5 of each individual) to compare AOR positions among individuals and across species.

### Comparison of 2D and 3D methods

We compared the accuracies of 2D (Reuleaux, 1876) and 3D methods using simulated rotations about AORs placed three distances posterior to the occiput: 0, 10 and 20 mm (Fig. 7). To simulate neurocranial motion during feeding, we created an animation in Maya containing a neurocranium (with anatomical landmarks) and a body plane. We applied known rotations to the neurocranium using Maya's keyframe tool, which allowed us to set the neurocranium's initial and final position. The intervening positions were interpolated by the software to produce a smooth motion from rest to peak neurocranial elevation. We performed two trials for each of the three AOR positions: one with planar motion of the neurocranium and one with non-planar 3D motion. Starting from a resting position, in the first trial (planar motion), the neurocranium was rotated only about a mediolateral axis, resulting in a total of 15° of neurocranial elevation. In the second trial (non-planar motion), the neurocranium again reached 15° of elevation from the same resting position but was also rotated with 7° of yaw (about a dorsoventral axis) and 3° of roll (about a anteroposterior axis).

We analyzed the simulated data using both the 2D Reuleaux method (Reuleaux, 1876; Chen and Katona, 1999) and the 3D JCS method used in this paper (Fig. 7). The Reuleaux method locates the AOR by tracking at least two landmarks with 2D kinematics (Fig. 7A). We tracked six landmarks that were arbitrarily placed on the neurocranium. We connected the starting (rest) and final (peak neurocranial elevation) positions of each landmark with a straight line. Finally, we drew perpendicular lines that bisected each straight line. The point of intersection between the bisecting lines is the estimated position of the AOR (Fig. 7A). The JCS method locates the AOR by quantifying relative motion between rigid bodies (Figs 2 and 7B). For the Reuleaux method, error was measured as the mean (±s.e.m) linear distance between the estimated and known AORs.

### Statistical analysis

Means and s.e.m. were calculated by taking the mean for each individual and then the mean of means, with sample size being the number of individuals. To test for inter-individual and interspecific differences in neurocranial rotations, we performed nested ANOVAs, with individuals nested within species. Means were calculated from the peak magnitudes attained during each strike for each of the three neurocranial rotations (i.e. elevation, yaw and roll). For yaw and roll, we ignored directionality (positive and negative) and instead calculated their means from their absolute values. Then we conducted Tukey post-hoc tests of pairwise species comparisons to test for significant differences between species. Statistical significance was declared at the 0.05 probability level, and all tests were performed using JMP Pro 12.2.0 (SAS Institute Inc., Cary, USA).
